# Leg Leiomyoma in Male Patients: A Report of Two Cases

**DOI:** 10.7759/cureus.78723

**Published:** 2025-02-08

**Authors:** Abdullah Zaher, Louise-Marie Mboua Tetka, Aïcha Ben Lakhdar, Noureddine Sekkach

**Affiliations:** 1 Orthopedics and Traumatology, Delafontaine Hospital, Saint Denis, FRA; 2 Pathology, Bichat Pathology Center, Paris, FRA

**Keywords:** angioleiomyoma, knee, leg, leiomyoma, musculoskeletal tumors

## Abstract

Leiomyomas, benign smooth muscle tumors, are most frequently found in the uterus. Leg leiomyoma has been reported in a small number of studies, making it an uncommon finding. We present two male patients who came to the clinic with bothersome masses in their calf and knee, respectively. The radiologists could not diagnose these masses during the preoperative workup.

According to pathology reports, the masses that were surgically removed were leiomyoma in one patient and angioleiomyoma in the other. Clinicians should consider leiomyoma when looking at soft tissue lumps, even though it's rare for men to have this condition in those areas. Clinical and radiographic confirmation of the diagnosis is challenging. However, a histologic analysis can support the diagnosis. The preferred course of treatment that provides instant symptom alleviation is surgical excision.

## Introduction

Benign smooth muscle tumors, known as leiomyomas, are mostly found in the uterus, impacting about 80% of women [1]. However, they may develop in other areas with smooth muscle, like the gastrointestinal tract [2]. The incidence of leiomyomas at the extremity level is higher in the lower extremities than in the upper extremities, although there is little evidence of these cases in the literature [3]. Based on the tissue of origin, leiomyomas can be further classified into three subtypes: genital leiomyomas, piloleiomyomas, and angioleiomyomas. The most prevalent subtypes of leiomyoma, angioleiomyomas and piloleiomyomas, can develop over the extremities [4].

Angioleiomyoma (also called vascular leiomyoma) arises from the muscular layer of the vessel wall. The most typical manifestation is a solid, painful swelling beneath the skin. Approximately 5% of all soft tissue tumors are angioleiomyomas [5]. Its subcutaneous location at the knee joint is rare, as is its location in the lower limbs [6]. It is more prevalent in women in third and sixth decades of their lives. Pregnancy, hormonal changes, infection, and traumatic venous congestion can be among the causes of vascular leiomyoma [7]. Since leiomyoma is an extremely uncommon diagnosis, a list of differential diagnoses can be made when a patient arrives at the clinic with any lower limb mass without mentioning leiomyoma as a potential diagnosis [3]. The diagnosis of leiomyomas is mostly based on histological characteristics because of their rarity and nonspecific clinical nature. This case report details the uncommon finding in two male patients who had two distinct leiomyoma subtypes removed from their legs.

## Case presentation

Case 1

The first patient was a healthy 31-year-old male who had a left calf mass that had been there for more than five years. According to the patient, the lump was smaller at first but has since slightly increased in size. He claimed that the contact irritates him, especially since he began participating in martial arts sports. He denied having related neurovascular symptoms when questioned. A green pea-sized, non-tender mid-calf mobile lump was palpable on the lateral side during physical examination. No distal neurovascular signs were present.

**Figure 1:**
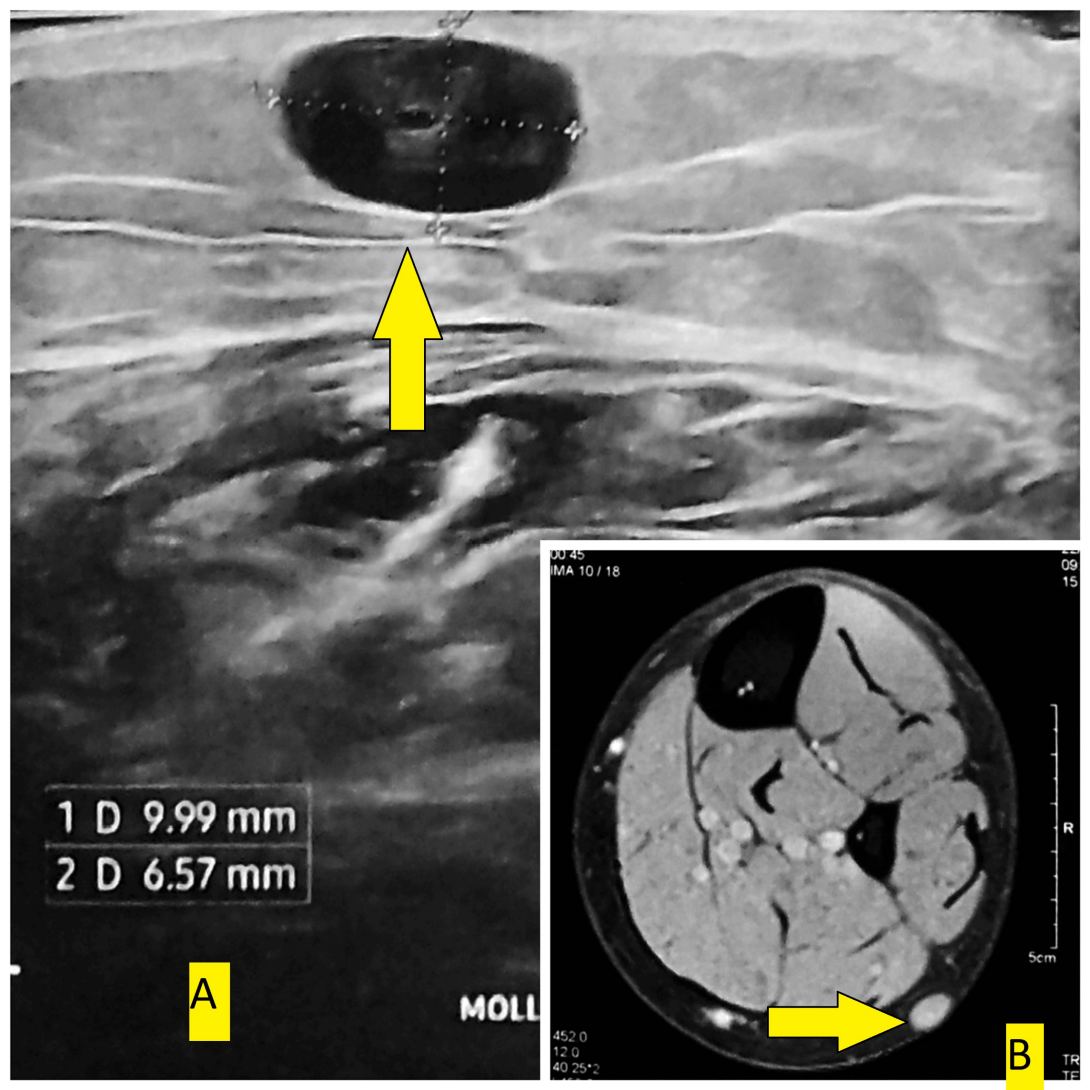
Preoperative scans of the first patient Preoperative echography (A) and MRI (B) images, with arrows marking the mass of interest.

On his initial visit, the patient brought an echography report that described a 10x5.6 mm subcutaneous lump on the lateral side, mid-height of the left leg, with central vascularization. According to the radiologist, the diagnosis was most consistent with adenopathy. An MRI was required to confirm the presence of a small non-specific subcutaneous nodule on the posterior calf measuring 9x6.6x1 mm, which is compatible with a cystic formation (Figure 1).

**Figure 2:**
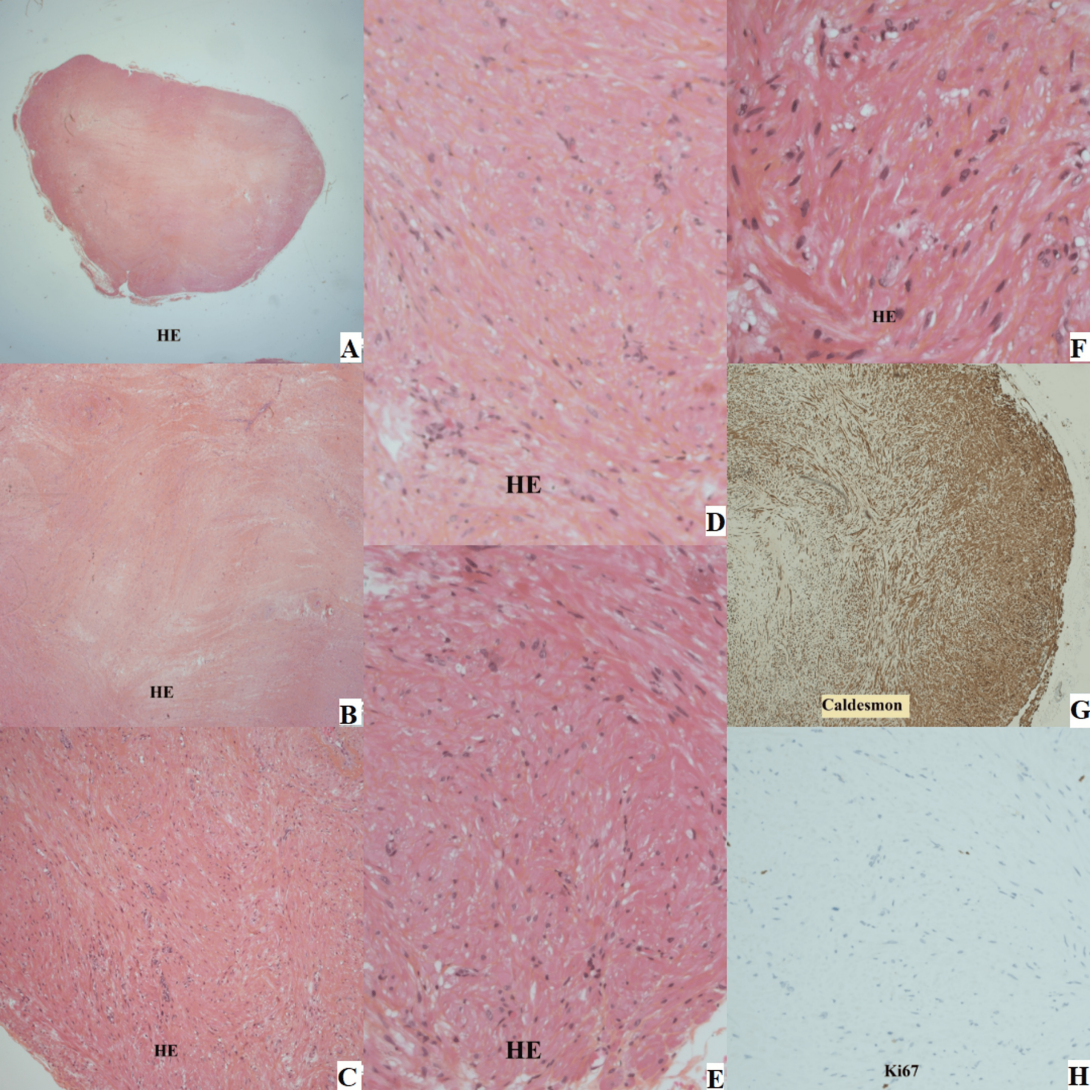
Histopathological study images (patient one) Histological slides of the excised tumor prepared with different stains  (A, B, C, D, E, F prepared with hematoxylin-eosin (HE); G prepared with caldesmon stain and H was ki67 marker stained ).

The patient was scheduled for surgical excision of this mass. During surgery, the mass was found just beneath the skin, easily excised, and sent for pathological analysis. With no indications of malignancy, the pathology report validated the histological diagnosis of leiomyoma (Figure 2).

Following surgery, the patient was seen at the clinic one month following the mass's removal. The patient's scar has healed. He was questioned and found to have no family history of uterine fibroids or malignancy, renal cell carcinoma, or other cancers. At the end of the visit, oral consent to publish the case was done.

Case 2

The second patient was a 48-year-old healthy man who presented with a mass in his right knee, which started appearing two years ago. The mass was localized to the anterior part of the knee just below the kneecap. It bothered him and was sometimes painful, yet it did not change in size with time. The lump was felt directly in front of the patellar tendon during the physical examination. It was spherical and about 1 cm in diameter.

**Figure 3:**
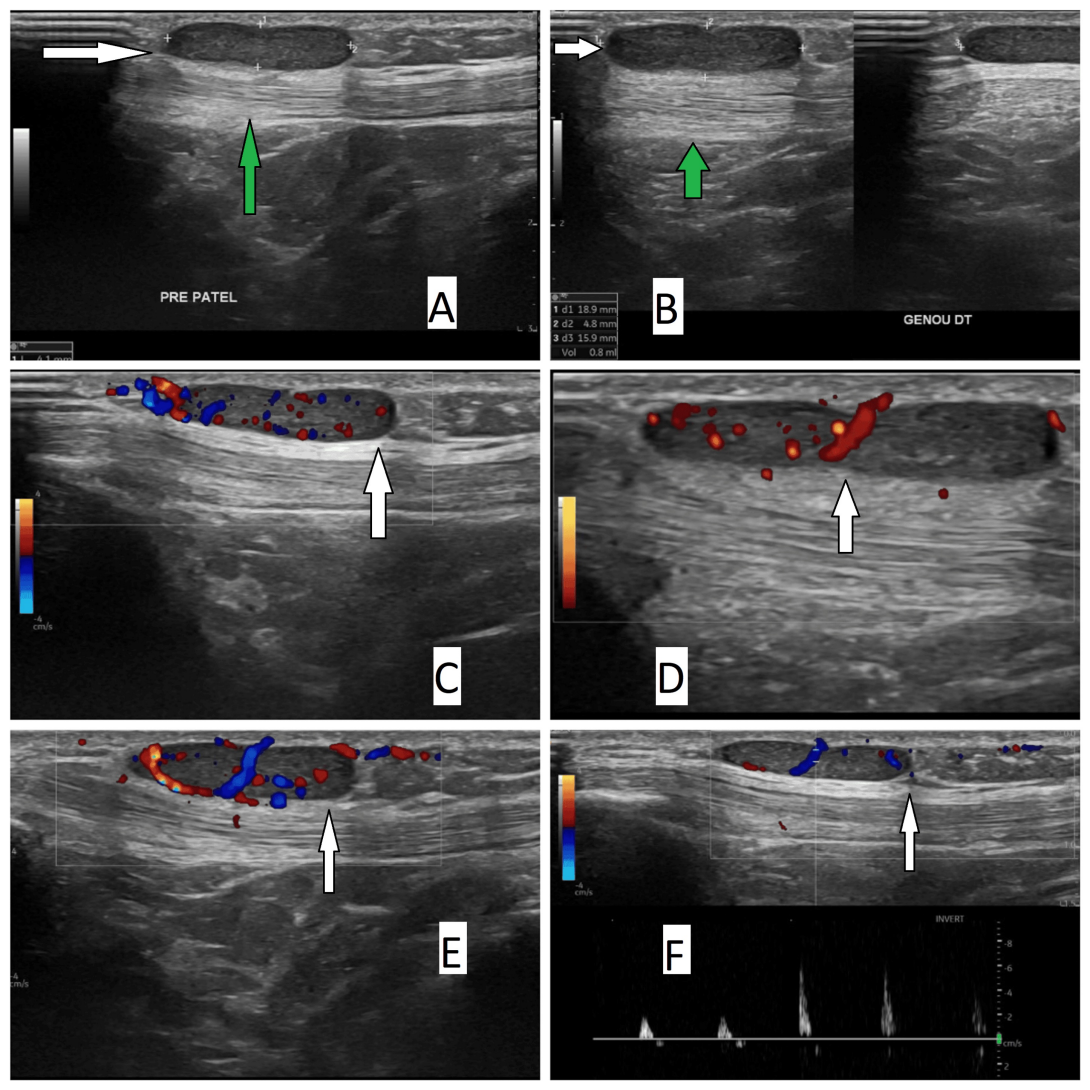
Preoperative scans of the second patient Preoperative echography (A, B) and echo-Doppler (C, D, E, F) images (white arrow: the mass, green arrow: the patellar tendon).

An echography was requested that revealed a well-defined, 4x17 mm pre-patellar subcutaneous mass that was vascularized on Doppler (Figure 3). The radiologist's differential diagnosis included vascular malformation or peripheral nerve tumor.

**Figure 4:**
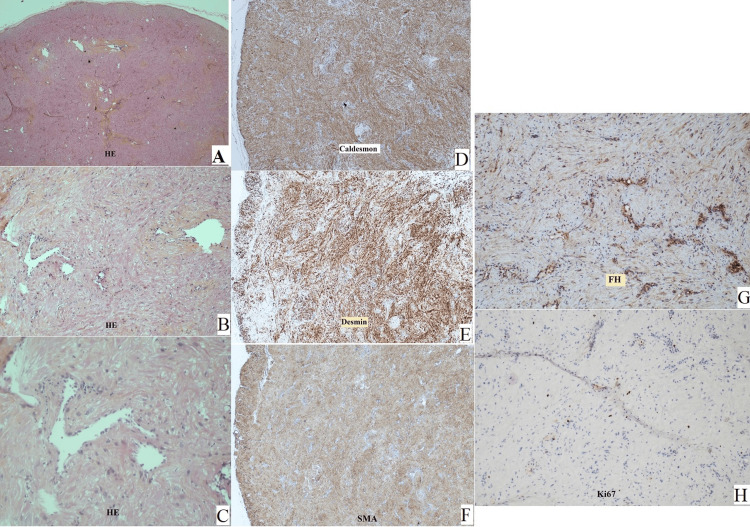
Histopathological study images (patient two) Histological slides of the excised tumor prepared with different stains (A, B, and C prepared with hematoxylin-eosin (HE) stain, D caldesmon stain, E desmin stain, F smooth muscle actin (SMA) stain, G fumarate hydratase (FH) stain, and H ki67 marker stain.

Surgical excision was programmed. The mass was easily excised and sent to pathological analysis. The pathology report confirmed the histological diagnosis of angioleiomyoma with no indications of malignancy (Figure 4).

Following surgery, the patient was seen at the clinic one month after the mass was removed. The patient's scar has healed. He was questioned again about his personal history of medical problems. He denied having any medical problems or any family history of malignancy. Before leaving the clinic, oral consent was obtained to publish the case.

## Discussion

Leiomyomas are benign tumors made of smooth muscle, mostly found in the uterus and digestive system. Cutaneous leiomyomas can be divided into three types: angioleiomyomas, piloleiomyomas, and genital leiomyomas. Angioleiomyomas and piloleiomyomas are the most common types of cutaneous leiomyomas, and they can show up on the arms and legs [4]. However, some studies showed variable distribution of these tumors with lower limbs affected in 9.3-43.4% of the cases [8].

These tumors, when located in the limbs, exhibit the same nonspecific symptoms as more common masses like lipomas and epidermoid cysts. The symptoms can vary depending on the location, size, and type of each mass. Additionally, paraclinical findings, such as ultrasound and MRI results, are often similar across these different types of masses. Clinical suspicion and diagnosis of such findings are difficult due to their rarity and lack of a diagnostic method to confirm the diagnosis other than histopathological examination [4].

Angioleiomyomas, also called angiomyomas or vascular leiomyomas, arise from the tunica media of veins. They make up 5% of all benign tumors in soft tissues. Factors like minor injuries, blood flow problems, and hormonal changes, especially estrogen, may affect their development. They are the type of cutaneous leiomyoma that most often appears in the extremities, with 60% presenting in the lower extremity below the thigh [9]. Typically, they show up as a small lump (usually less than 20 mm), and more than half of cases present with pain or tenderness. Most cases (around two-thirds) occur in people aged 40 to 60, and they are more common in women (2:1 ratio) [10]. 

In our second case, we reported a male patient in his fourth decade of life who manifested with a painful mass that was located just below the patella. Upon reviewing the literature, we found a similar case of a female patient of the same age presenting with a 0.5 cm nodule just below her kneecap that was eventually excised and turned out to be an angioleiomyoma [11].

Unlike angioleiomyomas, piloleiomyomas, also called pilar leiomyomas, come from the arrector pili muscle of the hair follicle. They often appear as firm, rounded nodules. Unlike angioleiomyomas, piloleiomyomas are usually not as painful. They typically develop in adolescents and younger adults, affecting both men and women equally [12]. In our first case, our patient was a young male presenting with a non-painful mass in his calf that was removed surgically and found to be a leiomyoma on microscopic analysis.

While reviewing the literature, there exists a syndrome of hereditary leiomyomatosis and renal cell cancer, also known as Reed’s syndrome. It is a rare autosomal dominant disorder caused by a mutation in the fumarate hydratase, a tumor suppressor gene, leading to an accumulation of fumarate. This syndrome is associated with benign smooth muscle tumors and an increased risk of renal cell carcinoma. In females, it is characterized by the presence of superficial leiomyomas besides uterine leiomyomas [13]. Thus, in the case of the presence of cutaneous leiomyomas, a clinician should be aware of this condition. The definitive diagnosis is made by genetic testing confirming the gene mutation [14]. However, there are certain diagnostic criteria that raise clinical suspicion for this syndrome. Major criteria include the finding of multiple skin lesions proven to be leiomyomas in at least one specimen, whereas the minor criteria consist of the discovery of a solitary cutaneous leiomyoma and family history of hereditary leiomyomatosis and renal cell carcinoma, early-onset renal papillary tumor, and multiple early-onset symptomatic uterine fibroids.

In our cases, none of the two patients was classified as having Reed's syndrome, as each had only one nodule, which was diagnosed as a cutaneous leiomyoma. In addition to this finding, which is considered a minor criterion, none of the other criteria applied to our patients. With respect to the treatment of these tumors, surgical excision leads to the absolute resolution of the symptoms, as shown in our cases.

## Conclusions

In summary, even though leiomyomas are not commonly found in the legs, they should still be considered in the list of the differential diagnosis of masses of lower extremities. Confirmation of the diagnosis is only achieved by having a microscopic analysis of such tumors. This means that removing the tumor through surgery helps not only in treating a patient’s symptoms but also in aiding in the diagnosis.

## References

[1] Eskenazi B, Warner M, Samuels S (2007). Serum dioxin concentrations and risk of uterine leiomyoma in the Seveso Women's Health Study. Am J Epidemiol.

[2] Lewis RB, Mehrotra AK, Rodriguez P, Levine MS (2013). From the radiologic pathology archives: esophageal neoplasms: radiologic-pathologic correlation. Radiographics.

[3] Zaher A, Yasser J, Badaro D, Sekkach N (2024). Unusual presentation of leiomyoma in the hindfoot. Case Rep Orthop.

[4] Buddemeyer K, McKissack HM, Farnell C (2018). Leiomyoma of the foot: a case report. Cureus.

[5] Hachisuga T, Hashimoto H, Enjoji M (1984). Angioleiomyoma. A clinicopathologic reappraisal of 562 cases. Cancer.

[6] Montemurro N, Ortenzi V, Naccarato GA, Perrini P (2020). Angioleiomyoma of the knee: an uncommon cause of leg pain. A systematic review of the literature. Interdisciplinary Neurosurgery.

[7] Santucci A, Albini M, Ventura A, De Palma L (2000). Clinical and histological features of vascular leiomyoma of the foot: case report and literature review. Foot Ankle Surg.

[8] Omoke NI, Aniume OI, Nwigwe CG, Utah FU (2024). Cutaneous leiomyoma of the leg: a case report and literature review. Sierra Leone J Med.

[9] Nishan B, Vivekanand Vivekanand, Vishnu M (2019). Vascular leiomyoma of the leg. Indian J Vasc Endovasc Surg.

[10] Kransdorf MJ, Jelinek JS, Moser RP Jr (1993). Imaging of soft tissue tumors. Radiol Clin North Am.

[11] Al Khalifa N, Falamarzi F, Al Hashimi H, Butt A (2022). Angioleiomyoma: a rare cause of anterior knee pain (case report). Bahrain Medical Bulletin.

[12] Kim DH, Lee JS, Kim JA, Lee JH (2017). Solitary piloleiomyoma in the scalp. Arch Craniofac Surg.

[13] Lehtonen HJ (2011). Hereditary leiomyomatosis and renal cell cancer: update on clinical and molecular characteristics. Fam Cancer.

[14] Patel VM, Handler MZ, Schwartz RA, Lambert WC (2017). Hereditary leiomyomatosis and renal cell cancer syndrome: an update and review. J Am Acad Dermatol.

